# Health literacy and health outcomes among older adults in Korea: a secondary analysis of 2023 KNHANES data

**DOI:** 10.1186/s12955-026-02597-7

**Published:** 2026-08-05

**Authors:** Hyunjeong Kwon, Eunhee Lee, Shin-Ae Kim, Jinhee Park, Jayun Kim

**Affiliations:** 1 College of Nursing, Keimyung University, Daegu, South Korea; 2 College of Nursing, Sungshin Women’s University, Seoul, South Korea; 3 College of Nursing, Seoul National University, Seoul, South Korea

## Abstract

**Background:**

Population aging has increased the prevalence of chronic diseases among older adults and increased the need for effective health information use. However, evidence regarding how health literacy is associated with health outcomes among older adults, and whether this association varies by chronic disease status, remains limited. Accordingly, this Korean study was conducted to evaluate the association between health literacy and health-related quality of life (HRQoL) and subjective health status among older adults.

**Methods:**

This study involved a secondary analysis of the data from the 2023 Korea National Health and Nutrition Examination Survey (KNHANES), a nationally representative cross-sectional survey. The sample included 1,568 adults aged ≥ 65 years. Health literacy was measured using the Health Literacy Index for the Community (HLIC) and classified as adequate (≥ 30) or inadequate (< 30). HRQoL was measured using the eight‑item Health-Related Quality of Life Instrument (HINT‑8), and perceived health status was dichotomized as good versus fair or worse. Multivariable linear and logistic regression models were used to examine associations between health literacy and health outcomes and to assess interaction effects with chronic disease status, adjusting for sociodemographic and health-related covariates.

**Results:**

Overall, 43.8% of participants demonstrated adequate health literacy. Adequate health literacy was associated with higher HRQoL (B = 0.023, *p* < 0.001), and higher odds of reporting good perceived health status (odds ratio = 1.57, 95% CI: 1.22–2.02). However, no statistically significant interaction between health literacy and chronic disease status was observed for either outcome, indicating no evidence of effect modification.

**Conclusions:**

The Korean study shows that health literacy is significantly associated with HRQoL and perceived health status. The pronounced gap in the technology and resources domain indicates a key vulnerability in the use of digital health information. Incorporating HL assessment into national health examinations and healthcare settings could help identify individuals with inadequate health literacy who may require prioritized educational support. At the healthcare system level, providing information and services tailored to patients’ health literacy levels may enhance their understanding, engagement, and ability to effectively manage their health.

**Supplementary Information:**

The online version contains supplementary material available at https://doi.org/10.1186/s12955-026-02597-7.

## Introduction

Population aging has emerged as a defining demographic trend of the 21st century, as the proportion of older adults is increasing rapidly worldwide [1]. While longevity represents a major public health achievement, it has increased the proportion of older adults living with multiple chronic conditions, functional decline, and age-related health vulnerabilities [2, 3]. These challenges often limit the ability of older adults to maintain independence and actively manage their health and thereby increase reliance on healthcare services and long-term care [3]. Consequently, promoting a longer life with healthier and more meaningful later years has become a critical societal concern. Furthermore, the health outcomes of older adults are being increasingly recognized as public health and social issues that affect healthcare systems, families, and communities.

Previous studies have identified numerous factors, such as sociodemographic characteristics (e.g. sex, income status, education, marital status, residential area), physical activity, chronic disease burden, functional status, and psychological conditions, associated with health outcomes [4–8]. While these factors are undoubtedly important, many are non-modifiable vulnerabilities inherent to aging, limiting their usefulness for practical strategies aimed at improving health outcomes. Furthermore, these factors provide only partial understanding of how health outcomes are formed in older adulthood. Even among individuals with comparable health problems, the management of personal health and engagement with health information and healthcare services vary considerably, contributing to variations in health outcomes [9]. These patterns suggest that health outcomes should be conceptualized as outcomes shaped by the ability of individuals to understand and manage personal health as well as the results of biomedical and demographic determinants.

Health literacy (HL) refers to the ability to access, understand, evaluate, and apply health-related information to facilitate decisions that maintain and improve health [10–12]. In recent years, research on HL has expanded, and many studies have reported that levels of HL among older adults are often inadequate [13, 14]. Given that a substantial proportion of older adults live with health problems and are required to understand and navigate complex health information and healthcare systems while managing these conditions, limited HL in this population remains a pressing concern. In particular, older adults with multimorbidity are exposed to high volumes of complex health information. Accordingly, HL may be particularly consequential in this population because they must integrate information across multiple conditions, treatments, and medications—often from different providers—while making ongoing management decisions. Moreover, ongoing changes in healthcare environments, such as the expansion of digital health services and reliance on technology-based health information, have placed additional demands on older adults regarding personal health comprehension and management [15, 16].

Although previous studies have suggested that HL is related to various health outcomes, such as self-management behaviour, healthcare utilization, subjective health status, and quality of life (QoL) [17–19], limited evidence exists regarding whether and how these associations vary according to the number of chronic conditions. Moreover, HL should not be viewed as a static attribute. Rather, it is a context-dependent capacity that may vary according to health status, healthcare demand, and evolving healthcare systems and health information [10–12]. The growing number of older adults living with health problems highlights the need for an examination of how HL influences health management and health outcomes among older adults within contemporary healthcare contexts.

Therefore, health problems among older adults have become an important issue that warrants societal attention. Although functional decline and an increased risk of health problems are inevitable aspects of aging [3], these age‑related challenges do not necessarily result in poorer health status or a reduced QoL. To prevent such outcomes, it is essential to strengthen the ability of older adults to understand health information and manage their health. However, although recent changes in healthcare environments have generally improved access to health information, these changes do not benefit older adults equally [16]. Moreover, increasingly complex health information and healthcare service systems pose additional challenges unless they are designed to be understandable and accessible [20]. Therefore, this study aimed to assess HL among older adults, and to examine its associations with health outcomes, including perceived health status and quality of life, per the number of chronic conditions; and investigate whether these associations differ by chronic disease groups.

## Materials and methods

### Study design and data source

This study involved a secondary analysis of data from the 2023 Korea National Health and Nutrition Examination Survey (KNHANES). KNHANES is a nationally representative, cross-sectional survey conducted regularly by the Korea Disease Control and Prevention Agency (KDCA) to assess the health and nutritional status of the general South Korean population. The KNHANES consists of three components: a health examination survey, a nutrition survey, and a health questionnaire. The KNHANES health survey used in this study was administered using a mixed-method approach, combining self-administered and interviewer-administered surveys. Specifically, interviewer-administered questionnaires were used for items such as the household survey, physician-diagnosed chronic conditions, and economic activity, among others. In contrast, self-administered questionnaires were used to assess items such as health literacy, alcohol consumption, and smoking.

The KNHANES collects health-related information annually from approximately 10,000 individuals aged ≥ 1 year, residing in about 4,800 households across 192 sampling units nationwide. A two-stage stratified cluster sampling design is employed, and survey districts are used as the primary sampling units and households as the secondary units. The sampling frame is based on data from the Population and Housing Census, and stratification is performed using city/province, residential area type (dong vs. eup/myeon), and housing type (general house vs. apartment). In the 2023 KNHANES, 6,929 of 9,825 eligible individuals participated, resulting in a participation rate of 70.5%.

### Study participants

Study participants were older adults aged 65 years or older adults. Respondents younger than 65 (*n* = 5,093) were excluded. respondents with missing data on variables were also excluded (*n* = 268). Consequently, 1,568 respondents were included in the analysis.

### Measurements

***Health literacy (HL).*** In the 2023 KNHANES, HL was assessed using the Health Literacy Index for the Community (HLIC), which was developed for the Korean population in 2022 [21]. The HLIC consists of 10 self-reported items (3 on disease prevention, 1 on health promotion, 4 on healthcare, and 2 on technology and resources), each item was rated on a 4-point Likert scale (1 = never, 2 = rarely, 3 = sometimes, 4 = always). Cronbach’s α at the time of development was 0.87 [21] and 0.91 in the present study. Originally, a cut-off score of 28 was proposed [21], but was later revised to 30 [22]. In this study, scores of ≥ 30 were classified as adequate and lower scores as inadequate.

***Health-related quality of life (HRQoL).*** In the 2023 KNHANES, HRQoL was assessed using the 8-item Health-Related Quality of Life Instrument (HINT-8), which was developed by the Korea Disease Control and Prevention Agency in 2014 for the general population in Korea. As HRQoL is influenced by cultural context, we considered the use of a Korea-specific instrument to be appropriate. The HINT-8 consists of eight items: climbing stairs, pain, vitality, working, depression, memory, sleep, and happiness, each with four response levels (1 = no problems, 2 = mild problems, 3 = moderate problems, and 4 = severe problems) [23]. The index value of the HINT-8 was derived from a general population–based preference study using both standard gamble and visual analogue scale methods [24]. The detailed procedure for calculating the HINT-8 index has been described in a previous study [24].

***Perceived health.*** Perceived health status was measured using a single question, “In general, how would you rate your health?” Five response options were provided (very good, good, fair, poor, very poor). For the analysis, responses were dichotomized as good (very good/good) versus other (fair/poor/very poor).

***Chronic disease groups.*** Chronic conditions, including hypertension, dyslipidaemia, diabetes mellitus, asthma, chronic sinusitis, allergic rhinitis, obstructive sleep apnoea, kidney disease, atopic dermatitis, and otitis media, were assessed based on self-reports. Responses were coded as 1 for “yes” and 0 for “no.” After summing the number of chronic conditions (range 0–10), we categorised the participants as having no chronic disease (0), a single chronic disease (1), or multimorbidity (≥ 2 chronic conditions).

***Sociodemographic and health-related covariates.*** We included sociodemographic and health-related covariates identified in previous studies as being associated with health outcomes. Sociodemographic characteristics included age (categorised as 65–74 years and 75 years or older), residential area (urban [dong] vs. rural [eup/myeon]), individual income level (low, low to middle, middle to high, and high), sex (male vs. female), education level (Elementary or less.

middle school, high school, college or higher), and marital status (married vs. other, including divorced, widowed, single). The health-related covariates were psychological health and health behaviors. Psychological health was assessed using perceived stress and anxiety levels. Perceived stress was assessed using a single item, “How much stress do you usually feel in your daily life?” Responses were rated on a 4-point scale (1 = very much, 2 = much, 3 = a little, 4 = hardly any). For the analysis, responses were dichotomized into high stress (very much/ much) and low stress (a little/hardly any). Anxiety was assessed using the Generalized Anxiety Disorder-7 (GAD-7). Scores were categorised as minimal (0–4), mild (5–9), moderate (10–14), and severe (15–21) [25]. We dichotomized the variable into a no-anxiety group (0–4) and an anxiety group (≥ 5). Alcohol use was coded as “yes” for participants who reported drinking alcohol at least once per month during the past year, whereas those who had never consumed alcohol or had consumed alcohol less than once per month during the past year were coded as “no.” Smoking status was coded as “yes” for individuals who had smoked at least five packs of cigarettes in their lifetime and who were current smokers; all others were classified as non-smokers. Regular physical activity was coded as “yes” for participants who reported engaging in at least 150 min per week of moderate-intensity physical activity, at least 75 min per week of vigorous-intensity physical activity, or an equivalent combination of moderate- and vigorous-intensity activity (1 min of vigorous activity was equated with 2 min of moderate activity). All others were coded as “no.”

### Statistical analysis

Data analysis was conducted using STATA/MP 18.0 (StataCorp, College Station, TX, USA). The analysis was conducted using sampling weights and a complex survey design to produce population-representative estimates. Categorical variables are presented as unweighted frequencies and weighted percentages according to HL groups. Item- and domain-level HL scores were analysed as weighted means, standard errors, and ranks according to HL group. Differences in the HINT-8 index and perceived health status according to HL groups were tested using adjusted Wald F-tests with weighted mean comparisons and Rao–Scott chi-square tests, reported as design-adjusted F statistics, respectively. Lastly, we conducted complex-sample multivariable logistic regression to examine the association between HL and perceived health status, and complex-sample multivariable linear regression to examine the association between HL and the HINT-8 index. To reduce the risk of overfitting, covariates were selected based on prior evidence. Multicollinearity was assessed using variance inflation factors. In Model 1, the associations between HL and health outcomes were estimated after adjustment for covariates. In Model 2, an interaction term between HL groups and chronic disease groups was additionally included to assess effect modification. All statistical tests were two-sided, with statistical significance set at *p* < 0.05.

### Ethical statement

The KNHANES 2023 survey was approved by the Institutional Review Board (IRB) of the Korea Disease Control and Prevention Agency (KDCA IRB No. 2022-11-16-R-A), and all participants provided written informed consent prior to participation. The present study is a secondary analysis of publicly available, fully de-identified KNHANES data from official website. As this study used existing data without collecting or recording personally identifiable information, it was considered exempt from IRB review in accordance with the Bioethics and Safety Act of Korea and its Enforcement Rule (Article 13, Paragraph 1, Item 3).

## Results

### General characteristics

Table 1 summarizes the general characteristics of the study participants. Participants’ mean age was 72.41 years, with 36.9% aged between 65 and 69 years. Among them, 864 (55.4%) were female, 1,132 (76.4%) resided in an urban area (dong), 702 (44.8%) had an education level of elementary school or below, and 1,118 (69.9%) were married. Regarding psychological characteristics, 235 participants (14.6%) reported experiencing a high level of stress. In addition, 173 participants (11.0%) were classified as having anxiety, defined as a GAD-7 score of 5 or higher. Regarding health behaviours, 546 participants (34.5%) reported drinking alcohol, 152 (9.5%) were current smokers, and 512 (33.2%) engaged in regular physical activity. Overall, 752 (48.4%) of older adults were classified as having multimorbidity, with a median of 1 (range: 0–8). Profiles of chronic diseases are shown in Supplementary File 1. Hypertension was the most prevalent condition (56.2%), whereas obstructive sleep apnea was the least prevalent (1.0%). Of the 1,568 participants, 43.8% were classified into the adequate HL group. The proportion with adequate HL was highest in the no chronic disease group (48.6%) and lowest in the multimorbidity group (two or more chronic diseases); however, this difference was not statistically significant.

### Health literacy profiles by the HL groups

A comparison of item- and domain-level mean scores of the two HL groups is provided in Supplementary file 2. Scores were lowest in the technology and resources domain and highest in the healthcare domain in both groups. However, the gap between groups was smallest in the Healthcare domain and greatest in the technology and resources domain. Item-level differences varied across the ten HLIC items. Among these items, the smallest gap between groups was observed for Q7 (understanding medication instructions), whereas the largest gap was observed for Q10 (applying online health information), which was followed by Q9 (evaluating online health information).

**Table 1:** General characteristics of participants (*N* = 1,568)

Variables		Total (*N* = 1,568)	Health literacy group		
			Adequate (*n* = 687, 43.8%)	Inadequate (*n* = 881, 56.2%)	Design-adjusted F (*p*)
Sociodemographic characteristics					
Age	65–69	590 (36.9)	285 (41.4)	305 (33.4)	11.87
	70–74	414 (24.0)	206 (28.3)	208 (20.6)	(< 0.001)
	75–79	345 (23.9)	129 (19.8)	216 (27.2)	
	80 or older	219 (15.2)	67 (10.5)	152 (18.8)	
Sex	Male	704 (44.6)	340 (49.7)	364 (40.6)	12.23
	Female	864 (55.4)	347 (50.3)	517 (59.4)	(< 0.001)
Residential area	Urban (dong)	1132 (76.4)	535 (82.3)	597 (71.9)	15.59
	Rural (eup/myeon)	436 (23.6)	152 (17.7)	284 (28.1)	(< 0.001)
Education	Elementary or less	702 (44.8)	217 (30.9)	485 (55.5)	48.77
	Middle school	324 (20.1)	136 (19.2)	188 (20.9)	(< 0.001)
	High school	354 (22.6)	202 (29.4)	152 (17.4)	
	College or higher	188 (12.5)	132 (20.5)	56 (6.2)	
Individual Income	Low	363 (22.1)	115 (16.0)	248 (27.0)	13.79
	Low to middle	388 (23.9)	151 (20.1)	237 (26.8)	(< 0.001)
	Middle to high	395 (25.2)	199 (28.6)	196 (22.4)	
	High	422 (28.8)	222 (35.3)	200 (23.8)	
Marital status	Married	1118 (69.9)	537 (77.6)	581 (63.9)	27.92
	Others	450 (30.1)	150 (22.4)	300 (36.1)	(< 0.001)
**Health-related characteristics**					
Regular exercise	No	1056 (66.8)	436 (62.7)	620 (70.0)	7.54
	Yes	512 (33.2)	251 (37.3)	261 (30.0)	(0.007)
Smoking	Smoking	152 (9.5)	56 (8.0)	96 (10.7)	2.90
	Non-smoking	1416 (90.5)	631 (92.0)	785 (89.3)	(0.091)
Alcohol use	Alcohol use	546 (34.5)	253 (37.2)	293 (32.3)	3.71
	No alcohol use	1022 (65.5)	434 (62.8)	588 (67.7)	(0.056)
Perceived stress	High stress	235 (14.6)	99 (13.5)	136 (15.5)	1.21
	Low stress	1333 (85.4)	588 (86.5)	745 (84.5)	(0.274)
Anxiety (GAD-7)	Anxiety (> 4)	173 (11.0)	60 (8.4)	113 (13.0)	6.75
	No anxiety (≤ 4)	1395 (89.0)	627 (91.6)	768 (87.0)	(0.010)
**Chronic Disease status**					
None		345 (21.4)	167 (23.8)	178 (19.6)	2.49
One chronic disease		471 (30.2)	212 (31.0)	259 (29.6)	(0.086)
Two or more chronic disease		752 (48.4)	308 (45.2)	444 (50.8)	

Note. Frequencies are raw values, and percentages are weighted

Abbreviations. HL, health literacy; SE, standard error; GAD, Generalized Anxiety Disorder-7

### Health outcomes by health literacy group

Table 2 presents HRQoL and perceived health status according to health literacy group. The overall mean HINT-8 index was 0.816. The mean HINT-8 index differed significantly between the inadequate and adequate HL groups, with values of 0.796 and 0.844, respectively (*p* < 0.001). Overall, 26.9% of participants reported their health as good or very good. The proportion of older adults reporting good or very good perceived health was significantly higher in the adequate HL group than in the inadequate HL group (34.0% vs. 21.6%, *p* < 0.001).

**Table 2:** Health-related quality of life (HINT-8 index) and Perceived health by health literacy group

Category	Total (*N* = 1,568)	Health literacy group		Design-adjusted F (*p*)
		Adequate HL (*n* = 687)	Inadequate HL (*n* = 881)	
HINT-8 index Mean (SE)	0.816 (0.003)	0.844 (0.004)	0.796 (0.004)	55.31 (< 0.001)
Perceived health (weighted % of good or very good)	26.9%	34.0%	21.6%	27.54 (< 0.001)

Note. All test statistics are reported as design-adjusted F values, accounting for the complex sampling design of KNHANES. Continuous variables were tested using adjusted Wald F-tests; categorical variables were tested using Rao–Scott chi-square tests, with test statistics converted to design-adjusted F values

### Multivariable associations between health literacy, perceived health, and HRQoL

Complex-sample multivariable linear regression analyses were used to examine the impact of HL on HRQoL and complex-sample multivariable logistic regression analysis was used to examine the association between HL and perceived health status. Variance inflation factors (VIF) were examined to assess multicollinearity. In the multivariable linear regression analysis for the HINT-8 index, VIF values ranged from 1.05 to 1.68. In the logistic regression analysis for perceived health status, VIF values ranged from 1.05 to 1.84. These values indicated no serious multicollinearity among independent variables.

The association of HL with HRQoL, as measured by the HINT-8 index, is presented in Table 3. In the adjusted model without interaction terms (Model 1), participants with adequate HL had significantly higher HINT-8 index scores (B = 0.023, *p* < 0.001). In Model 2, when an interaction term between health literacy and chronic disease status was examined, no significant interaction was observed for HINT-8 index. In Supplementary File 4, we examined the associations between HL and each HINT-8 item. Except for the pain item, adequate HL was significantly associated with HINT-8 items (Supplementary File 3B). The association between HL and perceived health status is shown in Table 4. In the adjusted model without interaction terms (Model 1), participants with adequate HL had higher odds of reporting good perceived health than those with inadequate HL (OR = 1.57, 95% CI: 1.22–2.02). In Model 2, when an interaction term between health literacy and chronic disease status was examined, no significant interaction was observed.

### Domain-specific associations of health literacy with perceived health status and health-related quality of life

In Supplementary File 4, domain-level HL analyses were conducted. The healthcare (B = 0.021, *p* = 0.004) and technology and resources (B = 0.017, *p* < 0.001) domains were significantly associated with higher HINT-8 index scores, whereas the disease prevention and health promotion domains were not. For perceived health status, no statistically significant associations were observed across domains; however, the health promotion domain showed a borderline association (OR = 0.80, 95% CI: 0.64–1.00).

**Table 3:** Multivariate linear regression analysis of the association between health literacy and health-related quality of life (HINT-8 Index)

Variables	Model 1			Model 2		
	B	SE	*p*	B	SE	*p*
**Adequate HL (vs. inadequate HL)**	0.023	0.006	< 0.001	0.028	0.012	0.020
**Chronic disease group (vs. none)**						
1	-0.003	0.008	0.727	0.0002	0.011	0.983
2+	-0.007	0.007	0.300	-0.004	0.009	0.690
**Interaction effect (vs. inadequate HL X no chronic disease)**						
Adequate HL X chronic disease (1)				-0.006	0.015	0.670
Adequate HL X chronic disease (2+)				-0.007	0.014	0.645
**Sociodemographic covariates -**						
Age (vs. 65–69 years)						
70–74	-0.010	0.006	0.108	-0.010	0.006	0.114
75–79	-0.015	0.008	0.065	-0.014	0.008	0.071
80 or older	-0.031	0.010	0.003	-0.310	0.011	0.004
Female (vs. male)	-0.026	0.006	< 0.001	-0.258	0.006	< 0.001
Urban area (vs. rural)	0.013	0.007	0.048	0.013	0.007	0.049
Education (vs. elementary or less)						
Middle school	0.013	0.009	0.123	0.013	0.009	0.122
High school	0.036	0.007	< 0.001	0.035	0.007	< 0.001
College or higher	0.038	0.010	< 0.001	0.038	0.010	< 0.001
Individual income (vs. low)						
Low to middle	-0.002	0.009	0.835	-0.002	0.009	0.831
Middle to high	0.026	0.007	0.001	0.026	0.007	0.001
High	0.014	0.008	0.093	0.014	0.008	0.096
Married (vs. others)	0.002	0.007	0.736	0.002	0.007	0.738
**Health-related covariates**						
Regularly exercise (vs. no)	0.017	0.005	0.002	0.017	0.005	0.002
Non-smoking (vs. smoking)	0.021	0.011	0.052	0.020	0.011	0.055
Non-Alcohol (vs. alcoholic)	-0.004	0.006	0.477	-0.004	0.006	0.467
Low stress (vs. high stress)	0.055	0.008	< 0.001	-0.055	0.008	< 0.001
No anxiety (vs. anxiety)	0.087	0.010	< 0.001	0.087	0.010	< 0.001

Note. All models were adjusted for age, sex, education level, chronic disease status, income, marital status, physical activity, smoking, alcohol use, perceived stress, and GAD-7 anxiety score; Model 1 included the main effects of health literacy and covariates; Model 2 additionally incorporated an interaction term between health literacy and chronic disease status

Abbreviations: B, unstandardized regression coefficient; HL, health literacy; SE, standard error; HINT, Health-related Quality of Life Instrument

**Table 4:** Multivariate logistic regression analysis of the association between health literacy and perceived health status

Variables	Model 1				Model 2		
	OR	95% CI			OR	95% CI	
**Adequate HL (vs. inadequate HL)**	1.57	1.22	2.02		1.28	0.73	2.24
**Chronic disease group (vs. none)**							
1	0.74	0.55	0.99		0.64	0.40	1.02
2+	0.60	0.44	0.81		0.52	0.34	0.80
**Interaction effect (vs. inadequate HL X no chronic disease)**							
Adequate HL X one chronic disease					1.33	0.65	2.73
Adequate HL X two or more chronic diseases					1.30	0.68	2.51
**Sociodemographic covariates**							
Age (vs. 65–69 years)							
70–74	0.97	0.68	1.37		0.75	0.56	0.99
75–79	1.21	0.85	1.71		1.37	0.99	1.90
80 or older	1.47	0.95	2.27		0.96	0.68	1.36
Sex (vs. male)	0.75	0.56	0.99		1.20	0.85	1.70
Residential area (vs. rural)	1.36	0.98	1.89		1.46	0.94	2.25
Education (vs. elementary or less)							
Middle school	1.29	0.89	1.85		1.28	0.89	1.85
High school	1.82	1.27	2.61		1.84	1.28	2.64
College or higher	2.67	1.71	4.17		2.66	1.70	4.15
Individual income (vs. low)							
Low to middle	1.33	0.94	1.89		1.33	0.93	1.90
Middle to high	1.15	0.78	1.70		1.16	0.78	1.72
High	1.08	0.74	1.59		1.09	0.74	1.61
Married (vs. others)	0.73	0.53	1.00		0.73	0.53	1.00
**Health-related covariates**							
Regularly exercise (vs. no)	1.43	1.12	1.83		1.43	1.11	1.83
Non-smoking (vs. smoking)	0.96	0.63	1.49		0.97	0.63	1.50
Non-Alcohol (vs. alcoholic)	0.88	0.67	1.16		0.88	0.67	1.16
Low stress (vs. high stress)	1.12	0.79	1.59		1.12	0.79	1.59
No anxiety (vs. anxiety)	2.88	1.64	5.04		2.90	1.65	5.08

Note. All models were adjusted for age, sex, education level, chronic disease status, income, marital status, physical activity, smoking, alcohol use, perceived stress, and GAD-7 anxiety score; Model 1 included the main effects of health literacy and covariates; Model 2 additionally incorporated an interaction term between health literacy and chronic disease status

Abbreviations: CI, confidence interval; HL, health literacy; OR, odds ratio

## Discussion

This study revealed an association between health literacy and health outcomes as determined using HRQoL and perceived health status after controlling for sociodemographic and health-related factors previously reported to be associated with HRQoL and health status in adults older than 65. Notably, participants with adequate HL reported higher HRQoL and had a greater odds of perceiving themselves as being in good health than those with inadequate HL. Furthermore, participants with adequate HL showed a 0.023-point higher HINT-8 index compared to those with inadequate HL. This difference remained statistically significant after adjustment for multiple covariates. Although the clinical relevance of this difference cannot be clearly determined due to the lack of an established minimal important difference for the HINT-8 index, the consistent association supports the role of health literacy as an important determinant of HRQoL. These findings align with previous findings that adequate HL appears to be linked with improved HRQoL and underscore the importance of HL as a determinant of health outcomes [10–12, 26]. Several potential mechanisms may explain the observed relationship between HL and subjective health outcomes. Health-literate older adults are more likely to understand medical information, navigate healthcare services and social support effectively, and engage in self-management behaviours such as adhering to treatment regimens, monitoring symptoms, and seeking timely care [27, 28]. These behaviours help them maintain a sense of control over their health and, in turn, enhance perceived health and HRQoL [29, 30].

Based on the interaction effect analysis, no statistically significant interaction was observed between health literacy and chronic disease groups. These results are consistent with previous research that also reported no significant interaction between chronic disease status and health literacy in relation to HRQoL [31]. These findings suggest that HL is an important factor associated with HRQoL among older adults regardless of chronic disease status, underscoring the importance of population-based HL assessment and management strategies. Previous studies have incorporated HL assessment into hospital and primary care settings to identify individuals with inadequate HL [32, 33] and this can support more effective communication between healthcare providers and patients with limited HL. However, healthcare-based screening may overlook older adults who have limited healthcare utilization or those without chronic conditions. Given that HL was associated with HRQoL regardless of chronic disease status in this study, population-based HL assessment should be considered to identify older adults with inadequate HL. In Korea, integrating HL assessment into the National Health Examination Program, which is conducted biennially among the general population, may provide an opportunity to identify individuals who require additional educational and supportive resources and reduce disparities in accessing, understanding, and applying health information.

Although HL was associated with HRQoL regardless of chronic disease status, HL may support health outcomes within different health contexts depending on individuals’ chronic disease status and healthcare demands. According to a recent systematic review suggests that the functional emphasis of HL may differ depending on health status [34]. Specifically, among individuals without chronic diseases, HL primarily functions as a resource that supports disease prevention and health promotion. Health-literate individuals are better equipped to engage in health-promoting behaviours, recognize early warning signs, and utilize preventive healthcare services effectively [35]. In contrast, older adults with chronic diseases face greater demands for processing and applying complex health information due to frequent healthcare interactions and disease management requirements [36, 37]. In this context, HL serves as important resource for effective disease management enabling individuals to interpret clinical information, adhere to complex treatment regimens, monitor symptoms, and communicate meaningfully with healthcare providers [27, 28].

Given that HL is a modifiable capacity that can be improved through interventions [38], efforts to enhance HL should be implemented among older adults regardless of chronic disease status. However, the focus and content of such interventions should be tailored considering individuals’ health conditions and healthcare needs. Among older adults without chronic diseases, HL enhancement may focus on supporting the maintenance of healthy lifestyles, recognition of health risk factors, and engagement in preventive health behaviors. Prior evidence suggests that HL-enhancing interventions are more effective when they incorporate multidimensional approaches, including multiple communication channels and active participation and discussion, rather than relying solely on one-way information delivery [39]. Therefore, integrating HL-sensitive components into existing community-based health promotion programs, along with opportunities for active discussion and communication among participants, may represent a practical strategy for improving older adults’ ability to access, understand, and use health information. In contrast, among older adults with chronic diseases, interventions should additionally support their ability to understand and apply health information necessary for effective disease management. Disease management interventions tailored to individuals’ HL levels have been shown to improve disease-related knowledge and self-management skills [40, 41], with literacy-matched education being particularly beneficial for individuals with lower HL [42]. Health literacy assessments, along with tailored information delivery and communication strategies, should be systematically integrated into chronic disease management at the hospital level.

Of the HL domains assessed, the technology and resources domain exhibited the greatest disparity between the adequate and inadequate HL groups and the lowest overall score. Moreover, this domain was significantly associated with HRQoL, which suggests that it may be a particularly important target for intervention. This finding is consistent with prior reviews identifying digital HL as a key determinant of quality of life [43]. Online platforms have become one of primary channels for health information delivery and increasingly shape how individuals seek and consume health-related content. However, digital sources such as social media and video-sharing platforms, like YouTube, commonly provide unreliable information [44, 45] because much of this content is designed to maximize viewership and often makes exaggerated claims. Furthermore, financial incentives or sponsorships may also contribute to the dissemination of biased information that prioritizes commercial interests over evidence-based guidance [46]. Older adults also tend to have lower levels of digital information processing skills [47], increasing their vulnerability to misinformation and its potential consequences for self-management and healthcare decision-making. These findings highlight the need for multifaceted interventions, including strengthening minimum regulatory measures for online health content, and education programs that enhance the tailoring of digital HL to the informational demands of older individuals with chronic diseases.

### Strengths and limitations

The present study has strengths and limitations that should be borne in mind when interpreting our findings. First, this Korean study used data from a nationwide representative sample of older adults (≥ 65), which enhances the generalizability of our results. In addition, because HL and HRQoL are likely to be influenced by cultural factors, we employed validated Korean-specific instruments to assess HL and HRQoL. However, several limitations must be acknowledged. First, owing to the secondary nature of the analysis, information on disease status was only available for the ten chronic conditions predefined in KNHANES. That is, we were unable to account for individuals exposed to other chronic diseases. Second, the HLIC includes a healthcare domain, which may limit its applicability among individuals with limited experience of healthcare utilisation. In addition, the HLIC was developed for the general population rather than specifically for older adults, which may affect its appropriateness for this age group. Older adults may have greater difficulty in comprehending item content. Moreover, individuals with lower HL may have been less likely to participate in the survey or to complete the self-administered instruments independently, which may have introduced selection bias and led to an underestimation of limited HL in the population. Additionally, the study relied on self-reported measures for key variables, which may be subject to recall bias and social desirability bias, potentially leading to measurement error. Furthermore, this study relied on secondary data collected using a complex sampling design; thus, the analyses were constrained by the variables available and the structure of the original survey. Lastly, the cross-sectional design of the study precluded access to temporal and causal relationships between HL and health outcomes. Future longitudinal studies are needed to clarify the nature of these associations and to examine potential causal pathways linking HL to HRQoL and perceived health status.

## Conclusion

This study shows that among older Korean adults, health literacy is significantly associated with HRQoL and perceived health status. The largest gap between health literacy groups was observed in the technology and resources domain and this domain was positively associated with HRQoL. Our results advocate that clinical practice and public health policy should move beyond standardized information provision by routinely assessing health literacy and delivering tailored health information and education. Furthermore, efforts should be strengthened to improve digital health literacy support, and measures are required to mitigate the impact of fragmented and conflicting health information.

## Supplementary Information

Below is the link to the electronic supplementary material.

Supplementary Material 1

## Data Availability

The data used in this paper are in the public domain and can be downloaded from https://knhanes.kdca.go.kr/knhanes/main.do#.

## Declarations

### Reporting guidelines

This study was conducted in accordance with the Strengthening the Reporting of Observational Studies in Epidemiology (STROBE) guidelines.

## Abbreviations

HINT-8: Health-Related Quality of Life Instrument with 8 Items
HL: Health literacy
HLIC: Health Literacy Index for the Community
HRQoL: Health-related quality of life
KDCA: Korea Disease Control and Prevention Agency
KNHANES: Korea National Health and Nutrition Examination Survey
