# Supplementary material for: Health literacy and health outcomes among older adults in Korea: a secondary analysis of 2023 KNHANES data
Source: Health Qual Life Outcomes. 2026 Aug 5;24:136. doi: 10.1186/s12955-026-02597-7 (PMC13729445; doi:10.1186/s12955-026-02597-7)
Supplement: Supplementary file 1 [file 12955_2026_2597_MOESM1_ESM.docx]

**Supplementary File 1. Chronic Disease Distribution Among Participants**

| **Chronic disease** | **Total** | **Health literacy** | |
| --- | --- | --- | --- |
|  |  | **Inadequate** | **Adequate** |
| Hypertension, n(%) | 875 (56.2) | 532 (60.6) | 343 (50.7) |
| Dyslipidemia, n(%) | 723 (46.1) | 408 (46.3) | 315 (46.0) |
| Diabetes mellitus, n(%) | 347 (22.0) | 199 (23.2) | 148 (20.5) |
| Asthma, n(%) | 72 (4.4) | 50 (5.6) | 22 (2.8) |
| Chronic sinusitis, n(%) | 82 (5.5) | 42 (5.4) | 40 (5.5) |
| Allergic rhinitis, n(%) | 146 (10.0) | 75 (9.1) | 71 (11.1) |
| Obstructive sleep apnea, n(%) | 13 (1.0) | 6 (0.9) | 7 (1.0) |
| Chronic kidney disease, n(%) | 57 (4.0) | 31 (3.8) | 26 (4.3) |
| Atopic dermatitis, n(%) | 19 (1.1) | 13 (1.3) | 6 (0.7) |
| Otitis media, n(%) | 68 (4.4) | 35 (3.7) | 33 (5.3) |

Note. Frequencies are raw values, and percentages are weighted.

**Supplementary File 2. Mean Scores and Rankings of Health Literacy Items by Health Literacy Group**

| **Domain** | **Item** | **Inadequate HL**  **(n=881)** | | **Adequate HL**  **(n=687)** | | |
| --- | --- | --- | --- | --- | --- | --- |
|  |  | **M (SE)** | **Rank** | **M (SE)** | **Rank** | |
| **Disease prevention** | Q1. Can you assess which vaccinations are necessary? | 2.48 (0.04) | 5 | 3.21 (0.02) | | 4 |
|  | Q2. Do you understand the degree of risk of mental health issues such as stress and depression? | 2.26 (0.04) | 8 | 3.14 (0.02) | | 8 |
|  | Q3. Are you aware of the health symptoms resulting from excessive drinking, smoking, and lack of exercise? | 2.33 (0.04) | 7 | 3.22 (0.02) | | 3 |
|  | **Mean** | **2.35 (0.03)** |  | **3.19 (0.02)** | |  |
| **Health promotion** | Q4. Can you evaluate how daily behaviors influence health? | 2.45 (0.04) | 6 | 3.20 (0.02) | | 5 |
| **Healthcare** | Q5. Do you have difficulty understanding explanations and instructions from doctors during medical appointments? | 2.73 (0.03) | 2 | 3.25 (0.02) | | 2 |
|  | Q6. Can you assess what needs to be prioritized in case of an emergency? | 2.52 (0.03) | 3 | 3.17 (0.02) | | 7 |
|  | Q7. Do you understand how to take medications as explained by doctors or pharmacists? | 2.85 (0.03) | 1 | 3.27 (0.02) | | 1 |
|  | Q8. Do you understand patient education materials provided by hospitals? | 2.49 (0.03) | 4 | 3.18 (0.02) | | 6 |
|  | **Mean** | **2.65 (0.03)** |  | **3.22 (0.02)** | |  |
| **Technology and resources** | Q9. Can you assess the reliability of health information obtained from the internet or media (e.g., television, YouTube)? | 2.17 (0.03) | 9 | 3.08 (0.02) | | 9 |
|  | Q10. Can you apply health information obtained from the internet or media (e.g., television, YouTube) to actual health-related behaviors or decision making? | 2.10 (0.03) | 10 | 3.07 (0.02) | | 10 |
|  | **Mean** | **2.13 (0.03)** |  | **3.07 (0.02)** | |  |

**Note**. 1 = never, 2 = rarely, 3 = sometimes, 4 = always; **Abbreviation.** HL, health literacy; M, mean; SE, standard error

**Supplementary File 3A. Mean Scores for Individual HINT-8 Items by Health Literacy Group**

**Note.** Each item is rated on a four-level scale, where score 1 indicates no impairment, and score 4 represents severe impairment.

| **Variable** | **Inadequate HL** | **Adequate HL** | **F(p)** |
| --- | --- | --- | --- |
| Climbing stairs | 1.99 (0.03) | 1.73 (0.03) | 37.56 (<0.001) |
| Pain | 1.94 (0.03) | 1.81 (0.03) | 12.22 (<0.001) |
| Vitality | 2.30 (0.04) | 1.90 (0.04) | 54.27 (<0.001) |
| Working | 1.94 (0.03) | 1.69 (0.03) | 33.32 (<0.001) |
| Depression | 1.55 (0.03) | 1.42 (0.02) | 13.89 (<0.001) |
| Memory | 1.87 (0.02) | 1.67 (0.02) | 35.34 (<0.001) |
| Sleep | 1.80 (0.03) | 1.65 (0.03) | 15.41 (<0.001) |
| Happiness | 2.40 (0.03) | 2.08 (0.04) | 34.80 (<0.001) |

**Supplementary File 3B. Adjusted Associations Between Health Literacy and Individual HINT-8 Items**

| **Independent variable** | **Dependent Variable** | **Multivariate regression model** | | |
| --- | --- | --- | --- | --- |
|  |  | **B** | **standard error** | ***p*** |
| Appropriate HL  (vs Inappropriate HL) | Climbing stairs | -0.109 | 0.040 | 0.007 |
|  | Pain | -0.206 | 0.037 | 0.579 |
|  | Vitality | -0.259 | 0.058 | <0.001 |
|  | Working | -0.095 | 0.041 | 0.020 |
|  | Depression | -0.088 | 0.031 | 0.005 |
|  | Memory | -0.157 | 0.033 | <0.001 |
|  | Sleep | -0.098 | 0.040 | 0.016 |
|  | Happiness | -0.223 | 0.057 | <0.001 |

**Note.** All models were adjusted for age, sex, education level, chronic disease status, income, marital status, physical activity, smoking, alcohol use, mental stress, and GAD-7 anxiety score.

**Abbreviations:** B, unstandardized regression coefficient; HL, health literacy;

**Supplementary File 4. Associations between Domain-level Health Literacy and Health Outcomes**

| **Variables** | **HINT-8 index^a^** | | | **Perceived Health Status^b^** | | |
| --- | --- | --- | --- | --- | --- | --- |
|  | **B** | **SE** | **p** | **OR** | **95% CI** | |
| **HLIC domain-level predictor** | | | | | | |
| **Disease prevention** | -0.007 | 0.007 | 0.331 | 1.29 | 0.95 | 1.75 |
| **Health promotion** | -0.005 | 0.007 | 0.437 | 0.80 | 0.64 | 1.00 |
| **Healthcare** | 0.021 | 0.007 | 0.004 | 1.08 | 0.72 | 1.63 |
| **Technology and Resources** | 0.017 | 0.004 | <0.001 | 1.20 | 0.99 | 1.46 |
| **Chronic disease group (vs. none)** | | | | | | |
| 1 | -0.003 | 0.008 | 0.695 | 0.73 | 0.54 | 0.98 |
| 2+ | -0.008 | 0.006 | 0.225 | 0.59 | 0.43 | 0.79 |
| **Socio-demographic covariates** | | | |  |  |  |
| Age (vs. 65-69) |  |  |  |  |  |  |
| 70-74 | -0.008 | 0.006 | 0.203 | 0.98 | 0.69 | 1.38 |
| 75-79 | -0.013 | 0.008 | 0.093 | 1.19 | 0.84 | 1.68 |
| 80+ | -0.029 | 0.010 | 0.007 | 1.48 | 0.95 | 2.31 |
| Sex (vs. male) | -0.024 | 0.006 | <0.001 | 0.75 | 0.57 | 1.00 |
| Residential area (vs. rural) | 0.012 | 0.007 | 0.107 | 1.36 | 0.97 | 1.91 |
| Education (vs. elementary or less) | | |  |  |  |  |
| Middle school | 0.011 | 0.009 | 0.184 | 1.27 | 0.88 | 1.83 |
| High school | 0.033 | 0.007 | <0.001 | 1.83 | 1.30 | 2.58 |
| College or higher | 0.037 | 0.009 | <0.001 | 2.76 | 1.78 | 4.28 |
| Individual income (vs. low) | |  |  |  |  |  |
| Low to middle | -0.004 | 0.009 | 0.682 | 1.30 | 0.91 | 1.86 |
| Middle to high | 0.026 | 0.008 | 0.001 | 1.16 | 0.79 | 1.71 |
| High | 0.012 | 0.008 | 0.151 | 1.08 | 0.74 | 1.58 |
| Married (vs. others) | 0.002 | 0.007 | 0.759 | 0.72 | 0.53 | 0.99 |
| **Health-related covariates** |  |  |  |  |  |  |
| Regularly exercise (vs. no) | 0.017 | 0.005 | 0.001 | 1.42 | 1.11 | 1.82 |
| Non-smoking (vs. smoking) | 0.021 | 0.010 | 0.043 | 0.96 | 0.63 | 1.48 |
| Non-Alcohol (vs. alcoholic) | -0.005 | 0.006 | 0.381 | 0.88 | 0.66 | 1.16 |
| Low stress (vs. high stress) | 0.053 | 0.008 | <0.001 | 1.12 | 0.78 | 1.59 |
| No anxiety (vs. anxiety) | -0.088 | 0.010 | <0.001 | 0.34 | 0.19 | 0.59 |

**Note.** The four HLIC domains (Disease prevention, Health promotion, Healthcare, and Technology and resources) were entered as separate predictors: a linear regression model for the HINT-8 index and a logistic regression model for perceived health status.

**Abbreviations:** B, unstandardized regression coefficient; CI, confidence interval; HL, health literacy; SE; HLIC, Health Literacy Index for the Community; SE, standard error; HINT, Health-related Quality of Life Instrument; OR, odds ratio.

**^a^** Multivariate regression analysis was conducted for HINT-8 index

**^b^** Multivariate logistic analysis was conducted for perceived health

**Supplementary file 5. Exploratory Subgroup Analysis of the Association Between Health Literacy and Health Outcomes**

| **Stratum** | **Category** | **HL group**  **(vs. inadequate)** | **HINT-8 index^a^** | | | **Perceived Health^b^** | |
| --- | --- | --- | --- | --- | --- | --- | --- |
|  |  |  | **B** | **SE** | **p** | **OR** | **95% CI** |
| Age | 65-69 | Adequate HL | 0.031 | 0.009 | <0.001 | 1.71 | 1.050-2.775 |
|  | 70-74 | Adequate HL | 0.008 | 0.011 | 0.460 | 1.50 | 0.912-2.453 |
|  | 75-79 | Adequate HL | 0.027 | 0.013 | 0.030 | 1.97 | 1.166-3.330 |
|  | 80 older | Adequate HL | 0.018 | 0.015 | 0.223 | 1.03 | 0.486-2.166 |
| Education level | Elementary or less | Adequate HL | 0.021 | 0.011 | 0.051 | 1.44 | 0.914-2.291 |
|  | Middle school | Adequate HL | 0.012 | 0.012 | 0.315 | 0.96 | 0.537-1.728 |
|  | High school | Adequate HL | 0.016 | 0.010 | 0.118 | 1.84 | 1.182-2.879 |
|  | College or higher | Adequate HL | 0.050 | 0.014 | <0.001 | 2.74 | 1.208-6.205 |

**Note.** All models were adjusted for age, sex, education level, chronic disease status, income, marital status, physical activity, smoking, alcohol use, mental stress, and GAD-7 anxiety score, except for the variable defining each stratum.

**Abbreviations:** B, unstandardized regression coefficient; CI, confidence interval; HL, health literacy; SE, standard error; HINT, Health-related Quality of Life Instrument; OR, odds ratio.

**^a^** Multivariate regression analysis was conducted for HINT-8 index

**^b^** Multivariate logistic analysis was conducted for perceived health
